# Retraction: Effects of rapid rehabilitation nursing model on surgical site wound infection and pain of patients with ovarian cancer: A meta‐analysis

**DOI:** 10.1111/iwj.70021

**Published:** 2024-08-09

## Abstract

Fan E, Zhang K, Wan Y, Hu S. Effects of rapid rehabilitation nursing model on surgical site wound infection and pain of patients with ovarian cancer: a meta‐analysi. *Int Wound J*. 2023; 21(3):e14464. 10.1111/iwj.14464

The above article, published online on 12 November 2023, in Wiley Online Library (http://onlinelibrary.wiley.com/), has been retracted by agreement between the journal Editor in Chief, Professor Keith Harding; and John Wiley & Sons, Ltd. It came to the publisher's attention from a third party that a number of articles shared concerning similarities in format and structure. Following an investigation by the publisher, the retraction of this article has been agreed on because the peer review and publishing process for this article were found to have been manipulated. The authors did not respond to our notice of retraction.

Fan E, Zhang K, Wan Y, Hu S. Effects of rapid rehabilitation nursing model on surgical site wound infection and pain of patients with ovarian cancer: a meta‐analysi. *Int Wound J*. 2023; 21(3):e14464. 10.1111/iwj.14464

The above article, published online on 12 November 2023, in Wiley Online Library (http://onlinelibrary.wiley.com/), has been retracted by agreement between the journal Editor in Chief, Professor Keith Harding; and John Wiley & Sons, Ltd. It came to the publisher's attention from a third party that a number of articles shared concerning similarities in format and structure. Following an investigation by the publisher, the retraction of this article has been agreed on because the peer review and publishing process for this article were found to have been manipulated. The authors did not respond to our notice of retraction.

